# Inter-eye Asymmetry in Refractive and Corneal Responses to Orthokeratology for Myopia Control in Rural Adolescents: A Paired-Eye Analysis

**DOI:** 10.7759/cureus.103312

**Published:** 2026-02-09

**Authors:** Bradley A Nordin

**Affiliations:** 1 Ophthalmology, Huffman and Huffman Eye Physicians and Surgeons, London, USA

**Keywords:** adolescents, anisometropia, appalachia, corneal curvature, inter-eye asymmetry, mean keratometry, myopia progression, orthokeratology (ortho-k), refractive error, rural eye care

## Abstract

Purpose

The purpose of this study is to evaluate inter-eye asymmetry in refractive error (spherical equivalent, SE) and corneal curvature (mean keratometry, Km) following orthokeratology (ortho-k) in adolescents, and to identify clinical predictors of asymmetric treatment response using a paired-eye design.

Methods

A retrospective analysis was conducted in 53 adolescents (106 eyes; ages 8-16 years) treated with orthokeratology for at least 6 months. Absolute inter-eye differences (oculus dexter - oculus sinister (|OD−OS|)) in SE and Km were calculated at baseline and post-treatment. Paired-samples t-tests assessed inter-eye differences, Pearson correlations evaluated bilateral symmetry, and linear regression models examined predictors of post-treatment asymmetry, including baseline asymmetry, age, sex, treatment duration, and interaction terms. Generalized estimating equations were used to evaluate predictors of annualized refractive progression while accounting for inter-eye correlation.

Results

Mean baseline inter-eye asymmetry was 0.28 ± 0.34 D for SE and 0.37 ± 0.29 D for Km. Following treatment, asymmetry remained low (ΔSE: 0.22 ± 0.23 D; ΔKm: 0.15 ± 0.32 D), with no significant differences between the right and left eyes. Bilateral correlations were strong for all refractive and corneal measures. A minority of participants demonstrated higher asymmetry exceeding commonly used clinical thresholds. Linear regression analyses identified no significant predictors of post-treatment asymmetry, with low overall model explanatory power. In contrast, older age at treatment initiation and longer treatment duration were associated with slower annualized myopia progression, while baseline asymmetry and sex were not.

Conclusions

Orthokeratology produces highly symmetric bilateral refractive and corneal responses in adolescents, with minimal inter-eye asymmetry and no identifiable predictors of asymmetric outcome. The consistency of treatment effects across eyes supports orthokeratology as a reliable and predictable myopia control strategy, including in pediatric populations managed in rural or underserved settings.

## Introduction

Myopia is an increasingly prevalent global public health concern, with projections estimating that nearly half of the world’s population will be myopic by 2050 and up to one billion individuals will be at risk for high myopia and its associated sight-threatening complications [1,2]. High myopia is strongly associated with retinal detachment, myopic maculopathy, and glaucoma, underscoring the importance of early intervention during childhood and adolescence [3,4]. Untreated myopia progression during these developmental years commonly averages approximately −0.50 diopters per year (D/y), with faster rates reported in younger children and certain demographic groups [5-7].

Orthokeratology (ortho-k) has emerged as an effective non-surgical intervention for myopia control, with multiple randomized controlled trials and meta-analyses demonstrating reductions in myopia progression and axial elongation of approximately 30-50% compared with single-vision spectacle correction [8-11]. The primary mechanism of ortho-k is believed to involve overnight corneal reshaping that induces relative peripheral myopic defocus, thereby altering retinal growth signaling and slowing axial elongation [12-14]. Long-term studies have demonstrated sustained efficacy with continued lens wear and an acceptable safety profile when appropriate hygiene and clinical follow-up are maintained [11,15,16].

Despite extensive evidence supporting the efficacy of ortho-k, the symmetry of treatment response between eyes has received comparatively little attention. Inter-eye refractive asymmetry is common in pediatric populations and has been associated with differential progression rates in untreated cohorts [6,17]. Most ortho-k studies analyze eyes independently or report group-level averages, which may obscure clinically relevant inter-eye differences in refractive or corneal response. Understanding whether ortho-k induces asymmetric treatment effects is therefore important for binocular visual development, lens fitting strategies, and long-term clinical management, particularly in children with baseline anisometropia.

This question is especially relevant in rural and underserved populations, where access to subspecialty eye care and frequent follow-up may be limited [18,19]. Appalachian communities, in particular, face persistent barriers related to geographic isolation, socioeconomic disadvantage, and reduced access to pediatric eye care services [20]. In a prior retrospective study, our group demonstrated that ortho-k significantly reduced myopia progression rates in adolescents from a rural Appalachian population compared with published untreated norms [21]. However, that analysis did not evaluate inter-eye asymmetry in refractive or corneal outcomes.

Accordingly, the purpose of the present study was to evaluate inter-eye asymmetry in refractive and corneal curvature responses following orthokeratology treatment in adolescents using a paired-eye design. The study aimed to quantify baseline and post-treatment asymmetry in spherical equivalent and mean keratometry, assess the magnitude and distribution of asymmetry, and identify clinical predictors of asymmetric response. It was hypothesized that orthokeratology would produce largely symmetric bilateral effects, even in the presence of baseline inter-eye differences.

## Materials and methods

Study design and population

This retrospective paired-eye study included adolescents treated with orthokeratology at a single rural clinical practice serving Appalachian communities [22,23]. Eligible participants were required to meet the following inclusion criteria: (1) age 8-16 years at the time of orthokeratology initiation; (2) bilateral orthokeratology lens wear; (3) a minimum treatment duration of six months; and (4) availability of complete refractive (spherical equivalent) and corneal topography (mean keratometry) data for both eyes at baseline and follow-up.

Exclusion criteria included the presence of ocular pathology other than refractive error (e.g., keratoconus, corneal dystrophy, active ocular surface disease), prior ocular surgery, history of amblyopia or strabismus requiring treatment, incomplete bilateral clinical data, or inconsistent orthokeratology lens wear that precluded reliable assessment of treatment response.

Fifty-three adolescents (106 eyes) met all inclusion criteria and were included in the final analysis. All participants were of White/Caucasian descent and resided in rural Appalachian communities. Demographic characteristics are summarized in Table 1.

**Table 1:** Summary characteristics of study participants OD: oculus dexter (right eye); OS: oculus sinister (left eye)

Participants	Number (n=53)	%
Male	27	51%
Female	26	49%
Eyes	106	
OD	53	50%
OS	53	50%
Ethnicity		
White, Caucasian	53	100%

Orthokeratology Treatment

All patients were fitted with Boston XO reverse-geometry rigid gas-permeable ortho-k lenses (Bausch + Lomb, Quebec, Canada) according to standard clinical protocols [9-11,15]. Lenses were worn overnight and removed upon waking. Follow-up visits included refraction and corneal topography assessments.

Outcome Measures

Primary outcomes included spherical equivalent refraction (SE), defined as the sphere power plus one-half of the cylindrical correction, consistent with standard refractive reporting [24], and mean keratometry (Km), defined as the average of the principal corneal curvature values (K1 and K2) representing overall central corneal curvature. Absolute inter-eye asymmetry was calculated as the absolute difference between the right (OD) and left (OS) eyes (|OD−OS|) for each variable at baseline and post-treatment. Annualized refractive progression rates were calculated when longitudinal data were available. For subject-level analyses, bilateral measurements were averaged within individuals to avoid pseudo-replication, consistent with best practices for paired-eye ophthalmic studies [25,26].

Statistical Analysis

Paired t-tests were used to compare OD and OS measurements at baseline and follow-up. Pearson correlation coefficients were used to assess bilateral symmetry. Linear regression models were used to evaluate predictors of post-treatment asymmetry, including baseline asymmetry, age, sex, treatment duration, and sex × asymmetry interaction. Generalized estimating equations (GEE) with an exchangeable correlation structure were used to model predictors of annualized refractive progression while accounting for inter-eye correlation [27].

Statistical analyses were performed using IBM SPSS Statistics (version 30.0; IBM Corp, Armonk, NY, US). A p-value < 0.05 was considered statistically significant.

## Results

Baseline characteristics

The mean age at treatment initiation was 11.2 ± 2.3 years (range: 8-16 years), with a mean orthokeratology treatment duration of 56 ± 31 months (range: 6-147 months). At baseline, the mean absolute inter-eye asymmetry was 0.28 ± 0.34 D for spherical equivalent and 0.37 ± 0.29 D for mean keratometry, consistent with previously reported ranges of inter-eye refractive variability in pediatric cohorts [6,17]. Baseline clinical parameters for study participants are summarized in Table 2.

**Table 2:** Baseline clinical characteristics of study participants Best corrected visual acuity (BCVA) for all patients in this study sample was 20/20; ^a^Initial refractive error, prior to treatment start, in spherical equivalents (SE), defined as sphere plus one-half the cylinder on cylindrical correction; ^b^Calculated as the absolute difference in refractive power (in spherical equivalents, SE) between the right (OD) and left (OS) eyes of an individual; ^c^Calculated as the absolute difference in mean keratometry (Km) between the right (OD) and left (OS) eyes of an individual; D: diopters

Clinical parameter	Mean ± SD	Minimum	Maximum
Age at baseline, years	11.2 ± 2.3	8	16
Treatment duration, months	56 ± 31	6	147
Baseline spherical equivalent (SE_i_)^a^, D	−2.10 ± 1.12	–7.5	–0.75
Baseline mean keratometry (Km_i_), D	43.21 ± 1.34	40.72	47.36
Baseline inter-eye SE asymmetry^b^, D	0.28 ± 0.34	0	1.88
Baseline inter-eye Km asymmetry^c^, D	0.37 ± 0.29	0.05	1.34

Inter-eye symmetry of refractive and corneal outcomes

No statistically significant differences were observed between right (OD) and left (OS) eyes for spherical equivalent or mean keratometry at baseline or following orthokeratology treatment (paired t-tests, all p > 0.05; Table 3). Bilateral correlations between eyes were strong for all refractive and corneal measures at both time points (Pearson ρ = 0.87-0.96, all p < 0.001; Table 4), consistent with prior reports of symmetric response to orthokeratology [14,28], and indicating a high degree of bilateral concordance in treatment response.

**Table 3:** Inter-eye asymmetry in refractive error and corneal curvature before and after orthokeratology Paired-samples t-tests were used to compare right-eye (OD) and left-eye (OS) measurements. Reported values include mean ± SD, t statistics, and corresponding p-values. D: diopters; OD: oculus dexter (right eye), OS: oculus sinister (left eye); SE: spherical equivalent refractive error (sphere plus one-half the cylinder on cylindrical correction)

Parameter	Baseline Asymmetry (|OD−OS|)	Post-Treatment Asymmetry (|OD−OS|)	t value	p-value
Spherical equivalent, SE (D)	0.28 ± 0.34	0.22 ± 0.23	0.88	0.38
Mean keratometry, Km (D)	0.37 ± 0.29	0.15 ± 0.32	1.06	0.29

**Table 4:** Bilateral correlations between eyes Pearson correlation coefficients (r) and corresponding p-values were calculated using Pearson’s correlation test to assess bilateral symmetry between the right (OD) and left (OS) eyes. Km: mean keratometry; OD: oculus dexter (right eye), OS: oculus sinister (left eye); SE: spherical equivalent refractive error (sphere plus one-half the cylinder on cylindrical correction)

Outcome Variable	Pearson r (OD vs OS)	p-value
Baseline refractive error (SE_i_)	0.91	< 0.001
Post-treatment refractive error (SE_f_)	0.96	< 0.001
Baseline mean keratometry (Km_i_)	0.87	< 0.001
Post-treatment mean keratometry (Km_f_)	0.93	< 0.001

**Figure 1:**
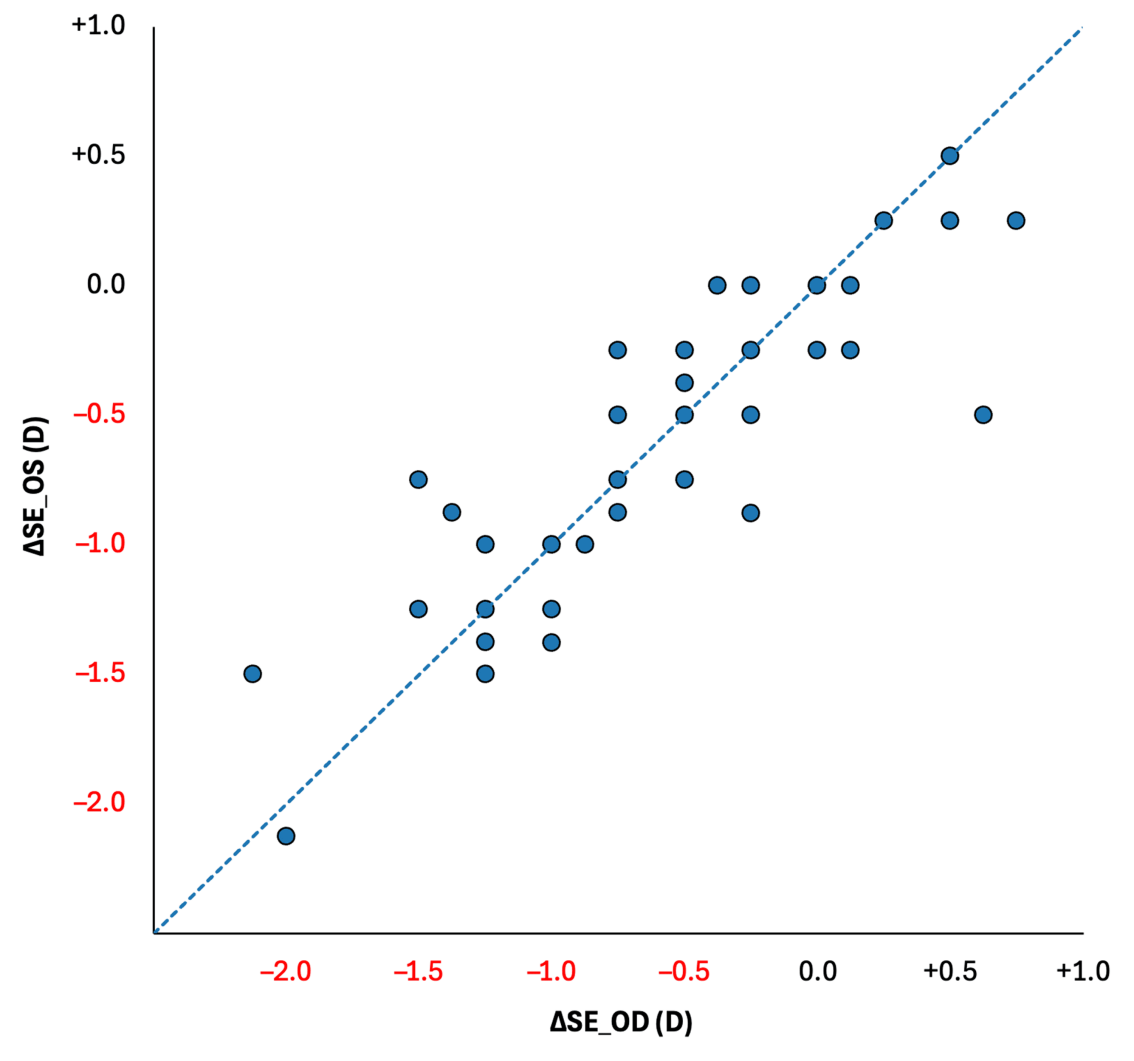
Comparing changes in spherical equivalent refraction (ΔSE) between right (OD) and left (OS) eyes following orthokeratology Each point represents one participant (n = 53). The diagonal line represents the line of identity (y = x), indicating equal refractive change between eyes. The close clustering of points along the identity line demonstrates strong bilateral symmetry in refractive response to treatment. D: diopters

A scatter plot comparing right-eye and left-eye changes in spherical equivalent (ΔSE) visually reinforces this bilateral concordance (Figure 1). Individual data points clustered closely along the line of identity, demonstrating that refractive changes induced by orthokeratology were highly symmetric between eyes across participants.

Inter-eye asymmetry magnitude and distribution

Post-treatment asymmetry in refractive and corneal outcomes was minimal. The mean absolute asymmetry in refractive change (|(ΔSE_OD − ΔSE_OS)|) was 0.22 ± 0.23 D, while the mean absolute asymmetry in corneal curvature change was 0.15 ± 0.32 D (Table 3).

**Figure 2:**
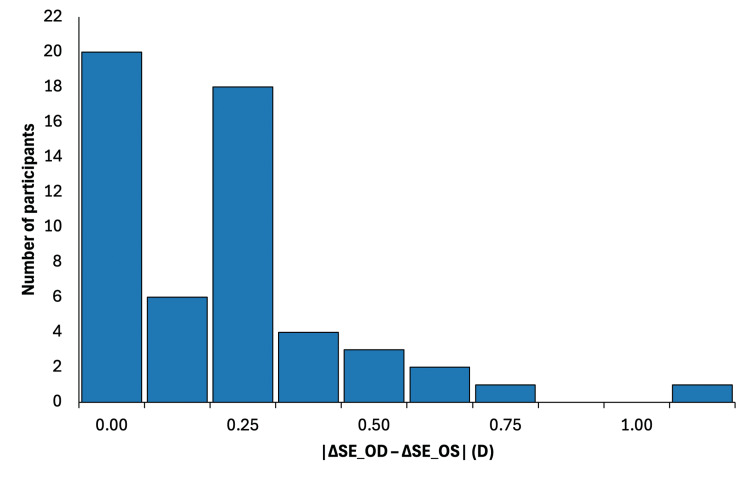
Distribution of absolute inter-eye asymmetry in refractive change following orthokeratology. Histogram showing the absolute difference between right-eye and left-eye changes in spherical equivalent (|ΔSE_OD − ΔSE_OS|). Most participants demonstrated minimal asymmetry, with relatively few exceeding commonly used clinical thresholds. D: diopters

Although most participants demonstrated low inter-eye asymmetry, a subset exhibited higher asymmetry exceeding commonly used clinical thresholds for refractive asymmetry. Depending on the threshold applied (0.5-0.8 D), 11-23% of participants met criteria for higher asymmetry, thresholds commonly cited in anisometropia and refractive literature [6,29]. The distribution of absolute inter-eye asymmetry in refractive change is shown in Figure 2, which demonstrates that the majority of participants clustered at low asymmetry values, with relatively few outliers.

Predictors of post-treatment asymmetry

Linear regression analyses revealed no significant predictors of post-treatment inter-eye asymmetry in refractive outcomes. Baseline SE asymmetry, age at treatment initiation, sex, treatment duration, and the sex × asymmetry interaction term were all non-significant predictors (all p > 0.15), with low explanatory power across models (R² < 0.07; Table 5). The absence of identifiable predictors of post-treatment asymmetry is consistent with prior work suggesting limited predictability of inter-eye refractive divergence over time [17].

**Table 5:** Predictors of post-treatment inter-eye asymmetry β coefficients, t statistics, and p-values derived from multiple linear regression models predicting post-treatment inter-eye asymmetry (Model R² = 0.074; F = 0.96, p = 0.439). SE: spherical equivalent refractive error

Predictor	β Coefficient	t value	p-value
Baseline SE asymmetry	0.144	1.45	0.153
Age at initiation	0.009	0.71	0.484
Sex	−0.034	−0.50	0.617
Treatment duration	−0.001	−1.06	0.294
Sex × asymmetry interaction	0.021	0.34	0.737

Myopia progression

Generalized estimating equation (GEE) analysis of annualized refractive progression demonstrated that increasing age at treatment initiation (B = −0.037, p = 0.021) and longer orthokeratology treatment duration (B = −0.005, p = 0.001) were significantly associated with slower myopia progression (Table 6). In contrast, baseline inter-eye asymmetry, sex, and interaction terms were not associated with progression rate (all p > 0.6).

**Table 6:** GEE analysis: predictors of annualized myopia progression Regression coefficients, Wald χ² statistics, and p-values were derived from generalized estimating equation (GEE) models with an exchangeable correlation structure accounting for inter-eye correlation. GEE: generalized estimating equation; SE: spherical equivalent refractive error

Predictor	B (SE)	Wald χ²	95% CI	p-value
Age	−0.037	5.33	−0.068 to −0.006	0.021
Treatment duration	−0.005	10.78	−0.008 to −0.002	0.001
Baseline SE asymmetry	0.004	0.08	−0.028 to 0.036	0.783
Sex	0.011	0.22	−0.036 to 0.058	0.636
Sex × asymmetry	−0.006	0.11	−0.041 to 0.029	0.737

## Discussion

The present paired-eye analysis demonstrates that orthokeratology produces highly symmetric bilateral refractive and corneal responses in adolescents, even in the presence of baseline inter-eye differences. Strong correlations between the right and left eyes and minimal absolute asymmetry following treatment suggest that ortho-k induces consistent optical and biomechanical effects within individuals. These findings extend prior evidence supporting ortho-k efficacy by showing that its myopia control benefits are not only effective but also predictably bilateral.

Previous studies have established the effectiveness of ortho-k in slowing myopia progression and axial elongation in children and adolescents [8-11,15,16]. However, most investigations have analyzed eyes independently or focused on population-level averages, which may obscure clinically meaningful inter-eye differences. By employing a paired-eye design, the present study directly addresses this gap and provides visual confirmation of bilateral concordance through scatter analysis of refractive change (Figure 1). The close clustering of points along the line of identity reinforces the statistical findings and supports the conclusion that treatment responses between eyes are closely matched.

Inter-eye asymmetry in refractive error is relatively common in pediatric populations and has been associated with differential progression rates in untreated cohorts [6,17]. Such asymmetry can raise clinical concerns regarding binocular vision development, lens-fitting strategies, and long-term refractive balance. In the current study, although a subset of participants exhibited asymmetry exceeding commonly cited clinical thresholds, the overall distribution of asymmetry was skewed toward low values (Figure 2), and higher asymmetry was not associated with poorer outcomes or accelerated progression. This pattern suggests that observed variability likely reflects normal biological heterogeneity rather than inconsistent treatment effects.

The absence of identifiable predictors of post-treatment asymmetry further supports the robustness of ortho-k treatment. Neither baseline refractive imbalance, age, sex, nor treatment duration significantly predicted asymmetric response. These findings are consistent with emerging biomechanical evidence indicating that ortho-k induces relatively uniform corneal epithelial redistribution and curvature changes across eyes, even when baseline corneal geometry differs modestly [14,22,23]. This uniformity likely reflects the dominant role of peripheral retinal defocus in modulating ocular growth rather than localized baseline refractive differences [12,13].

Importantly, generalized estimating equation analysis demonstrated that older age at treatment initiation and longer treatment duration were associated with slower myopia progression, while baseline inter-eye asymmetry was not. These results align with prior longitudinal studies showing that younger age is a key risk factor for faster progression and that sustained ortho-k wear is necessary to maintain treatment efficacy [15,16,24]. The lack of association between asymmetry and progression further suggests that clinicians need not alter treatment strategies solely based on baseline inter-eye differences.

From a clinical perspective, the demonstrated bilateral consistency of ortho-k is particularly relevant for rural and underserved populations. In settings where access to subspecialty care and advanced imaging may be limited, treatment modalities that offer predictable and symmetric outcomes can simplify clinical management and patient counseling [18-20]. This is especially important in pediatric patients, for whom long-term adherence and continuity of care are critical. Our findings build upon prior work showing effective myopia control in rural Appalachian adolescents treated with ortho-k and further support its use as a reliable strategy in such populations [21].

Limitations

Several limitations of this study should be acknowledged. First, the retrospective design introduces inherent constraints, including potential selection bias and reliance on the completeness and accuracy of existing clinical records. Although all eligible patients meeting predefined inclusion criteria were included, data availability limited analyses to refractive and keratometric outcomes routinely collected in clinical practice. Axial length measurements and detailed corneal biomechanical parameters were not consistently available and therefore could not be evaluated.

Second, data extraction was restricted to visits with complete bilateral measurements for both spherical equivalent and mean keratometry; records lacking paired-eye data, incomplete follow-up, or inconsistent orthokeratology lens wear were excluded to ensure analytic validity. While this approach strengthens internal consistency, it may limit generalizability to broader clinical populations. Finally, the study cohort was demographically homogeneous and derived from a single rural clinical practice, which may further limit external applicability. Despite these limitations, the paired-eye design, extended treatment duration, and use of statistical methods accounting for inter-eye correlation support the robustness of the findings.

## Conclusions

In conclusion, this paired-eye analysis demonstrates that orthokeratology produces highly consistent and symmetric bilateral refractive and corneal curvature responses in adolescents, even in the presence of baseline inter-eye differences. Absolute inter-eye asymmetry in spherical equivalent and mean keratometry remained low following treatment, and no clinically meaningful differences were observed between right and left eyes. Importantly, commonly considered patient and treatment factors, including age at initiation, sex, treatment duration, and baseline asymmetry, did not predict asymmetric outcomes, indicating that treatment effects were robust and uniform across individuals. These findings suggest that orthokeratology exerts predictable bilateral effects on both refractive error and corneal shape, supporting its reliability as a myopia-control strategy in pediatric populations. From a clinical perspective, the demonstrated symmetry of treatment response may simplify lens fitting, monitoring, and patient counseling, particularly in rural or underserved settings where access to frequent subspecialty follow-up may be limited. Overall, the results reinforce orthokeratology as a dependable and consistent intervention for myopia management in adolescents.
